# Metabolic Parkinson’s disease

**DOI:** 10.3389/fnagi.2025.1665957

**Published:** 2025-10-10

**Authors:** Federica Invernizzi, Lorenzo Ciocca, Elena Contaldi, Donato Inverso, Daniela Calandrella, Francesco Mignone, Michela Barichella, Ioannis Ugo Isaias, Gianni Pezzoli

**Affiliations:** ^1^Center for Liver disease, Division of Internal Medicine and Hepatology, IRCCS Ospedale San Raffaele, Milan, Italy; ^2^Vita-Salute San Raffaele University, Milan, Italy; ^3^Parkinson Institute of Milan, ASST G.Pini-CTO, Milan, Italy; ^4^Division of Immunology, Transplantation, and infectious Diseases, IRCCS San Raffaele Scientific Institute, Milan, Italy; ^5^Fondazione Pezzoli per la Malattia di Parkinson, Milan, Italy; ^6^Clinical Nutrition Unit, ASST G.Pini-CTO, Milan, Italy; ^7^University Hospital of Würzburg and Julius Maximilian University of Würzburg, Würzburg, Germany

**Keywords:** Parkinson’s disease, metabolic sybdrome, diabetes, Obesity, GLP-1/GIP RA

## Abstract

Parkinson’s disease (PD) is a neurodegenerative disorder primarily characterized by the loss of dopaminergic neurons in the substantia nigra. While most cases are sporadic, there is growing evidence of a link between PD and metabolic dysfunctions such as type 2 diabetes mellitus, obesity, and metabolic syndrome. Proposed pathogenic mechanisms underlying this overlap include insulin resistance and chronic inflammation. Similar patterns of cellular damage are observed in both metabolic disorders and PD, including mitochondrial dysfunction, impaired autophagy, oxidative stress, endoplasmic reticulum stress, and gut microbiota alterations. Given the current lack of disease-modifying therapies for PD, there is increasing interest in interventions traditionally used to treat metabolic conditions, such as lifestyle and dietary modifications. Notably, antidiabetic drugs like metformin and incretin mimetics have shown beneficial effects in PD due to their neuroprotective and anti-inflammatory properties, their ability to restore insulin sensitivity, and their role in reducing neuronal susceptibility to toxic insults, as demonstrated in both preclinical and clinical studies. Conversely, traditionally antiparkinsonian drugs such as bromocriptine have long been approved for improving glycemic control in diabetes. This cross-efficacy between drugs used for the two conditions may indirectly support the hypothesis of a shared pathogenesis. A deeper understanding of the connections between metabolic disorders and PD could pave the way for novel preventive and therapeutic strategies.

## 1 Introduction

Parkinson’s disease (PD) is the second most common chronic neurodegenerative disorder (NBD) after Alzheimer’s disease (AD), with a progressive loss of motor, cognitive, and autonomic (de Rijk et al., 2000; Poewe et al., 2017). The major pathological hallmarks of PD are represented by the progressive loss of dopamine(DA)-ergic neurons in the substantia nigra (SN) of the central nervous system (CNS) associated with cytoplasmic inclusions termed as Lewy bodies and Lewy neurites (Baba et al., 1998; Spillantini et al., 1997).

**FIGURE 1:**
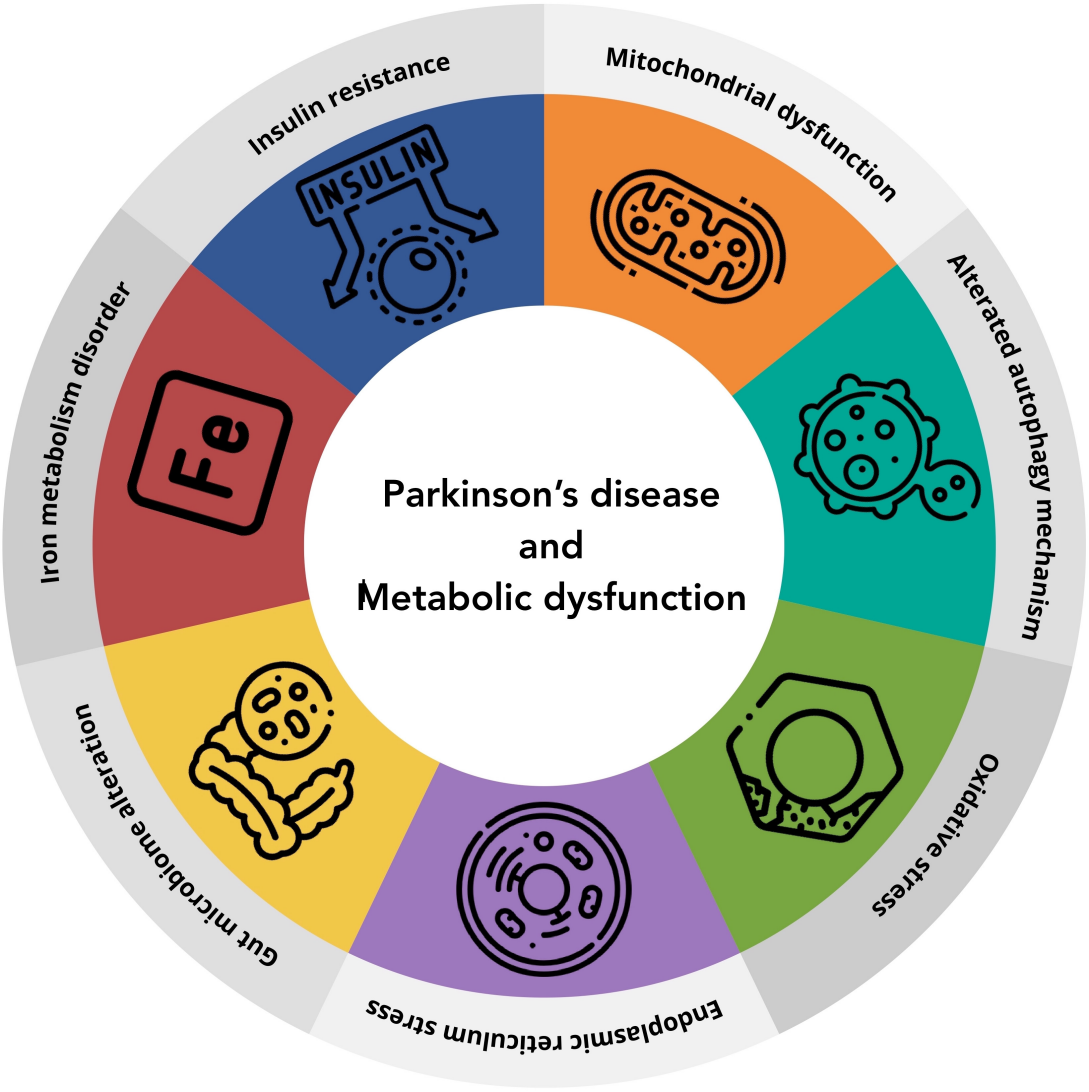
Schematic representation of the shared pathogenic mechanisms that underlie both PD and metabolic dysfunction.

In the spectrum of parkinsonian disorders, attempts to classify disease subtypes based on cluster of clinical features have been made (Marras and Lang, 2013), as well based on etiology. Some cases are sustained by genetic variants whereas other forms are idiopathic. Among monogenic variants for example, *GBA1*-associated PD is increasingly recognized and studied as an entity with earlier onset and rapid progression (Skrahin et al., 2024). Although the proportion of PD patients harboring pathogenic genetic variants is significant, with estimates ranging from 5%–15% to over 40% (Lim et al., 2024), most cases are sporadic. It is therefore assumed that diverse genetic, epigenetic, and environmental factors interact in a complex manner to affect disease risk and progression (Gundogdu et al., 2021; Roverato et al., 2021; Sircar et al., 2021).However, the mechanisms underlying the onset of sporadic PD remain unclear, with aging being a primary risk factor (Gundogdu et al., 2021). Interestingly, epidemiological, preclinical, and clinical data suggest an association between Type 2 diabetes mellitus (T2DM) and an increased risk or accelerated progression of PD signs and symptoms (Ribarič, 2024; Stockmann et al., 2025), with motor symptoms being especially affected (Chen et al., 2025; Kotagal et al., 2013). This supports the idea that insulin resistance (IR) and chronic inflammation contribute to the overlapping aetiologies of T2DM and PD. Insulin may play a key role in many processes that could be dysregulated in both diseases, including apoptosis, autophagy, mitochondrial dysfunction, oxidative stress, neuroinflammation, and synaptic plasticity (Cullinane et al., 2023; Yan et al., 2023; Figure 1). Furthermore, obesity and metabolic syndrome (MetS) also appear to be risk factors for the occurrence of PD through the same mechanism, namely systemic inflammation, insulin and leptin resistance, blood-brain barrier disruption, and altered brain metabolism (Neto et al., 2023; Table 1). Although these correlations are now known and consolidated, the exact mechanisms underlying the aetiopathogenesis and linking mechanisms remain unclear and data are heterogeneous. Future studies are therefore needed, especially to target prevention and subsequent treatment of PD through lifestyle interventions and the use of drugs with metabolic action. Indeed, the gold-standard therapy for the symptomatic treatment of PD is dopamine replacement therapy (DRT) with levodopa and other dopaminergic agents. Nonetheless, DRT does not address the underlying neurodegenerative processes that characterize PD and levodopa treatment can associate with motor fluctuations and dyskinesias, which largely impact the quality of life. Additionally, non-motor issues such as cognitive impairment and dysautonomia, pose major challenges with limited therapeutic strategies available.

**TABLE 1:** Table of shared pathogenic mechanisms in PD and metabolic dysfunction.

PD and metabolic dysfunction pathogenic overlap	Mechanism	Potential therapeutic targets
Gut microbiome alteration	Systemic inflammation, increased gut permeability, promotion of BBB disruption	Probiotics, prebiotics, fecal microbiota transplantation
Iron metabolism disorder	Cellular oxidative damage, ferroptosis, neurodegeneration, fibrosis deposition	Iron chelators, antioxidants
Mitochondrial dysfunction	Energy deficit (decreased ATP production), increased ROS	Inhibition of Drp1; NAC
Oxidative stress	Endothelial dysfunction, neuronal and glial damage, myelin reduction	Antioxidative compounds; NAC
Altered autophagy mechanism	Damaged protein and organelles accumulation, α-synuclein aggregation	Metformin (AMPK activity potentiation, mTOR activity reduction)
Endoplasmic reticulum stress	Protein misfolding and accumulation, UPR activation	UPR modulators
Insulin resistance	Metabolic and synaptic dysfunction	Metformin, GLP-1/GIP-RA

BBB, blood-brain barrier; ATP, adenosine triphosphate; ROS, reactive oxygen species; UPR, unfolded protein response; Drp1, dynamin-related protein-1; NAC, N-Acetylcysteine; AMPK, adenosine monophosphate-activated protein kinase; mTOR, mechanistic target of rapamycin; GLP-1/GIP-RA, glucagon-like peptide-1/glucose-dependent insulinotropic polypeptide receptor agonists.

In this context, our review aims to explore the possible existence of a form of PD that could be referred to as “metabolic PD,” given how tightly metabolic dysfunctions and NBD are growingly appearing to be connected in the latest scientific evidence, therefore requiring a multidisciplinary approach. The rising prevalence of metabolic dysfunction associated diseases worldwide is so concerning that experts have started to refer to it as a “pandemic” to highlight the need for enhanced awareness, dedicated public health screening and interventional programs. In addition, this is also promoting efforts to better comprehend the implication of the association between metabolism and NBD on the use of both traditional and emerging drugs generally used for T2DM and MetS to also treat conditions that up to now were only therapeutically approached from a neurologist’s perspective.

## 2 Metabolic mechanisms that may explain links between obesity, MetS, T2DM, and PD

It is currently accepted that neuroinflammation and oxidative stress are major factors in PD-related dopaminergic neuron degeneration (Chakrabarti and Bisaglia, 2023; Hassanzadeh and Rahimmi, 2018). T2DM and obesity are grouped in MetS, a complex disorder characterized by at least three of the following criteria: abdominal adiposity, IR, hypertension, hypercholesterolemia, and hypertriglyceridemia (Després and Lemieux, 2006; Monserrat-Mesquida et al., 2020). MetS promotes peripheral oxidative stress, low-grade chronic peripheral inflammation, and dysregulation of the adipose and circulating Renin–Angiotensin System (RAS) toward its pro-oxidative pro-inflammatory axis (Monserrat-Mesquida et al., 2020).

Interestingly, IR, inflammation, mitochondrial dysfunction, endoplasmic reticulum (ER) stress, autophagy, and the ubiquitin–proteasome system (UPS), independently contribute to the onset and aetiopathogenesis of both T2DM and PD. In obesity, circulating fasting glucose levels were found to be associated with the extent of brain atrophy and amyloid accumulation (Honea et al., 2022; Kueck et al., 2023). Inflammatory cytokines like Tumor necrosis factor (TNF)-α, chronically released in obesity, activate pathways that can lead to IR. Drugs targeting TNF-α or its pathway, like infliximab and etanercept, showed mixed results in improving insulin sensitivity, with some success observed over longer treatment periods (Kueck et al., 2023; Lyngsø et al., 2002; Matulewicz and Karczewska-Kupczewska, 2016; Stanley et al., 2011). Pharmacologic treatment of PD has also been a target of study for its role in metabolic glucidic homeostasis, as discussed below. The involvement and importance of DA as a neurotransmitter and neuromodulator which regulates CNS function are well known, but its effect on glucose homeostasis and pancreatic cell function are still not completely defined. Its effect is mediated through D2 receptors (D2Rs) in the brain, pancreas and pituitary gland thus regulating appetite, circadian rhythms, insulin and prolactin release (Lopez Vicchi et al., 2016).

### 2.1 Basic science research

Investigation of diet impact on PD with research using animal models reveals that high-fat diets worsen nigrostriatal DA depletion in the brain and exacerbate neurotoxicity (Griffioen et al., 2013). *N*-methyl-4-phenyl-1,2,3,6-tetrahydropyridine (MPTP) and 6-hydroxydopamine (6-OHDA) are neurotoxins used to induce animal models of PD. Studies of MPTP-induced and 6-OHDA-induced murine models of PD show greater striatal DA depletion in mice fed a high-fat diet compared to chow-fed animals. This depletion was significantly correlated with insulin resistance, as measured by the Homeostasis Model Assessment for Insulin Resistance (HOMA-IR) index (Bousquet et al., 2012).

Obesity-induced IR correlates with increased DA depletion in the striatum and substantia nigra, and additionally, it can affect both the postsynaptic DA receptor and presynaptic DA transporter expression and function (Hölscher, 2020). Preclinical studies revealed that obesogenic diets facilitate disease processes in mice harboring PD-related α-synuclein mutations. In a study using A53T mice, a high-calorie diet exacerbated autonomic dysfunction that occurs in early disease stages in these animals. This effect was ameliorated by intermittent energy restriction (Griffioen et al., 2013). These findings were extended to motor decline and mortality in the A30P α-synuclein mouse model of PD. When fed a high-fat diet, these mice became insulin-resistant and exhibited earlier motor decline and death than their standard chow-fed counterparts (Rotermund et al., 2014).

It is reported that pharmacological treatment of PD with DRT plausibly plays a role on glucose metabolism, with the finding that in animal model levodopa and DA can suppress glucose-dependent pancreatic islets secretion by reducing the frequency of intracellular calcium current oscillation, thus representing a DA-ergic negative feedback on insulin secretion (Ustione and Piston, 2012). An additional connection between PD and glucose metabolism derives from the observation of the role of bromocriptine: a DA agonist active on D2 historically used for PD, which was later approved by Food and Drug Administration to treat T2DM (Pijl et al., 2000). Several preclinical studies demonstrated that administration of bromocriptine to diabetic or obese animal models improved glucose intolerance, reduced high insulin and lipid levels in serum. The bromocriptine action is in part related to hypothalamic DA circadian rhythm modification and corticosteroid rhythms reset. Furthermore, in spontaneously hypertensive rats with metabolic syndrome bromocriptine impacted on liver metabolism potentiating a reduction of elevated lipogenic and gluconeogenic capacity. Bromocriptine directly activates α2-adrenergic receptors and inhibits glucose stimulated insulin secretion in pancreatic β cells (Cincotta et al., 1989, 1999; Ezrokhi et al., 2014). Studies using conditional mutant female mice lacking D2Rs in pituitary lactotropes (lacDrd2KO) also point to an indirect role of DA on glucose homeostasis. These mice display chronic hyperprolactinemia and lactotrope hyperplasia. As a result, increased food intake, marked body weight gain and adipose tissue accretion, as well as glucose intolerance develop (Perez Millan et al., 2014).

### 2.2 Clinical studies

Curiously, an epidemiologic, large-scale, population-based study on 6,800,000 individuals from the database of the Korean National Health Insurance Service observed that being underweight may be a risk factor for the development of PD. An increased risk of PD incidence was observed in the underweight group versus the normal BMI group (adjusted hazard ratio, aHR: 1.28; 95% CI: 1.21–1.36). Conversely, obese patients showed a decreased risk of PD incidence (aHR, 0.90; 95% CI: 0.88–0.93 in the obese group and aHR: 0.77; 95% CI: 0.72–0.82 in the severely obese group). In the analysis stratified by T2DM status, considering the higher risk of PD in the T2DM group, these associations were robust in those with T2DM, including an increased risk in underweight individuals and a decreased risk in obese individuals. The authors hypothesized a neuroprotective effect of hyperinsulinism in obese diabetic patients which could be attributed to counteracting the decrease in cell-surface gamma-aminobutyric acid receptors, reduction of glutamate, and through the regulation of the Phosphoinositide 3-Kinase (PI3K) pathway. However, the research was conducted solely on the Korean population whose metabolic characteristics related to BMI and T2DM could be different from those of other ethnicities, thus requiring further evaluations (Jeong et al., 2020).

Metabolic dysfunction-associated steatotic liver disease (MASLD), and metabolic dysfunction-associated steatohepatitis (MASH) are common chronic liver conditions that often present with MetS. In the context of MetS, liver diseases and NBD share common mechanistic pathophysiologic features which led to investigation for a link between MASLD/MASH and extrahepatic manifestations such as neurodegeneration. Multiple studies have been trying to establish an association between metabolic liver diseases and neurodegenerative disorders, although a definite bidirectional causal link has not been identified. Cognitive impairment, defined as an intermediate stage between the normal aging process and the onset of dementia, has a multifaceted etiology but specifically in the setting of metabolic disorders is sustained by a spectrum of factors, prominently featuring IR, lipotoxicity, and vascular-systemic inflammation, which are also shared with MASLD. The brain-gut liver axis with the intricate connections of neural, immune, and endocrine systems, also seems to play a role. The severity of cognitive impairment seems likely to progress with the individual burden of liver fibrosis and a higher incidence of white matter lesions was found in MASH compared to non-MASLD patients (Medina-Julio et al., 2024). Shang et al. (2021) proved through liver biopsy that MASLD by the histological grade of fibrosis, can predict the risk of dementia. In a recent prospective study on a sample of 1600 patients, MASLD was linked to cognitive deterioration, particularly in middle-aged people (Liu et al., 2021). In another prospective study on 2800 patients, the prevalence of NAFLD was related to worse cognitive function (Gerber et al., 2021). As for PD in the context of NBD, available data are not sufficient to establish an association. A sex-stratified analysis by van Kleef et al. (2023). on almost 9000 Dutch patients ruled out an association between fatty liver disease defined by ultrasound or FLI (Fatty Liver Index), and PD. A previous analysis by Jeong et al. (2021) on 2,000,000 patients from the Korean National Health Insurance Service detected, amongst men who developed PD in the follow-up period, a decreased risk of PD in those with MASLD defined by FLI [aHR: 0.86, 95% confidence interval (CI): 0.82–0.91] and in contrast, among women, an increased risk in those with MASLD (aHR: 1.09, 95% CI: 1.02–1.16). With MASLD affecting around 30% of the global population, further investigation is needed in this field to provide clearer evidence and strategies for early detection and management of both liver disease and neurologic impairment.

Quite large evidence is available to support the correlation between T2DM and PD (Aune et al., 2023; Chohan et al., 2021; Park et al., 2023).However, little is known about the correlation of PD with Type 1 Diabetes (T1DM). A recent multivariable Mendelian randomization (MVMR) analysis by Wang et al., exploring the potential protective effect of T1DM on the risk of PD in individuals of European ancestry, is worth mentioning. Interestingly, genetic susceptibility prediction of T1DM was found to be correlated with a lower risk of developing PD (OR = 0.9708; 95% CI: 0.9466, 0.9956; *P* = 0.0214) (Wang Q. et al., 2024). This observation is further supported by an epidemiologic investigation conducted by Senkevich et al. (2023).

As investigated on animal models, drugs for PD were also studied in human for their effect on glucose homeostasis. Various studies were previously undertaken to evaluate the effect of bromocriptine on weight and glycaemia in obese non-diabetic and diabetic individuals, and marked reductions in fasting plasma glucose levels were found. In addition, weight loss, body fat loss and improved glucose tolerance was also reported in clinical trials in obese men, as well as cardiovascular benefits (Chamarthi et al., 2015; Garber et al., 2013). Bromocriptine also presents a beneficial effect on plasma lipids probably secondary to the activation of CNS DA-ergic pathways. It significantly reduces both lipolysis and lipogenesis but as the reduction is greater for lipogenesis the net effect is to promote fat mobilization and reduce fat storages (Liang et al., 2015).

### 2.3 Insulin resistance

Insulin regulates cell growth, gene expression, protein synthesis, mitochondrial function, and autophagy. It also regulates the activation of neural stem cells, cognition, synaptic formation, and neuronal apoptosis. Implications of neuronal insulin resistance are commonly seen in various neurodegenerative diseases (Ahmad et al., 2019; Bosco et al., 2012; Hölscher, 2020; Hong et al., 2020).

#### 2.3.1 Basic science research

Evidence from *in vitro* and *in vivo* studies reports that insulin can affect both postsynaptic DA receptor and presynaptic DA transporter expression and function (Anitha et al., 2012; Jones et al., 2017). Insulin acts as a ligand and binds to its receptor resulting in enhanced tyrosine kinase activity that phosphorylates insulin receptor substrate (IRS)-1 and IRS-2 that bind to p85-phosphati-dylinositol 3-phosphate kinase (PI3K) phosphorylating Akt and glycogen synthase kinase-3beta (GSK3-β), thereby regulating glucose metabolism (Hölscher, 2020). Dysregulation of the PI3K/Akt signaling pathway may alter the expression of α-synuclein, leading to DA-ergic cell death. It is also of note that glycated insulin has an increased propensity to aggregate. Studies have also highlighted the role of leucine-rich repeat kinase 2 (LRRK2) in insulin-dependent intracellular signaling. Reports suggest that phosphorylation of Ras-related protein (Rab10), an endocytic sorting protein by LRRK2 is crucial for glucose transporters type 4 (GLUT4) translocation to the neuronal plasma membrane. This process is inhibited in PD patients with the LRRK2 (G2019S) mutation (Athauda and Foltynie, 2016; Sekar and Taghibiglou, 2018).

#### 2.3.2 Clinical studies

Wang et al. recently investigated the role of hyperglycemia by dividing PD patients into high- and low-glycated hemoglobin (HbA1c) groups. The study found that PD with higher HbA1c had worse motor symptoms, particularly axial symptoms like slower gait speed and postural control issues. Higher HbA1c levels were associated with worsened automatic walking, while fast walking speed was not affected by hyperglycemia. This suggests that hyperglycemia primarily impacts automatic motor functions controlled by the basal ganglia rather than cortical-controlled activities. Moreover, hyperglycemia was linked to balance issues, but IR did not appear to play a significant role in this. The authors speculate that hyperglycemia affects postural control through mechanisms beyond IR, possibly by damaging vestibular and cholinergic pathways. Interestingly, while some studies suggest that hyperglycemia worsens cognitive function, no significant difference in cognitive function was found between high and low HbA1c groups in this study (Wang R. et al., 2024). In addition, the role of glucose metabolism impairment was observed in a meta-analysis encompassing more than 1,700,000 patients, finding diabetic patients to have a 38% higher risk of development of PD (Yue et al., 2016), while in another meta-analysis on 86,000 PD patients, prediabetic patients were found to have a 4% higher risk of development of PD compared to individuals with no fasting glucose impairment (Aune et al., 2023).

### 2.4 Mitochondrial dysfunction

Mitochondria are crucial for insulin secretion from pancreatic β cells, and their dysfunction contributes to impaired insulin secretion in T2DM. Mitochondrial DNA (mtDNA) depletion and mutations in certain mitochondrial genes are linked to reduced insulin secretion and increased oxidative stress, although studies on the exact mechanisms are scarce (Lev et al., 2006).

#### 2.4.1 Basic science research

Similarly, in the context of PD, mitochondrial dysfunction can drive neurodegeneration. Neurons are particularly susceptible to mitochondrial damage due to their high metabolic and energetic request. One of the main molecular pathologic mechanisms in this setting is represented by α-synuclein aggregation in mitochondria, which leads to the production of reactive oxygen species (ROS) and induces activation of p38 MAPK, a MAP-kinase that triggers the phosphorylation of Drp1 (dynamin-related protein-1), which eventually leads to mitochondrial fission (Filichia et al., 2016). Genetic damage in Parkin, PTEN Induced Kinase 1 (PINK1) genes and in the DJ-1 protein (which acts as a neuroprotective redox sensor) result in decreased activity of mitochondrial complex I enzyme and decreased adenosine triphosphate (ATP) production (Greco et al., 2023; Lev et al., 2006). Overexpression of the LRRK2 gene leads to decreased mitochondrial membrane potential and ATP levels (Beal, 2003; Martin et al., 2014).

Therefore, both T2DM and PD show strong connections between mitochondrial damage and increased oxidative stress, resulting in altered insulin secretion in the former case, and in neuronal damage and neuroinflammation in the latter.

### 2.5 Altered autophagy mechanism (and role of GBA1 gene)

Autophagy is a process that in normal conditions maintains cellular balance by recycling proteins and organelles, and acts as an adaptive mechanism that helps cells survive under stressful conditions.

#### 2.5.1 Basic science research

In T2DM, impaired autophagy in pancreatic β cells leads to the accumulation of metabolic products and damaged organelles (Xxxx et al., 2022). Increased LC3-II levels indicate enhanced autophagic activity. In T2DM, elevated LC3-II and p62 levels suggest that autophagic flux is active but may be dysfunctional due to inefficient degradation of the autophagosomes. Another key role in the biogenesis of autophagy and autophagosome formation is played by Atg7 and the Atg12-Atg5 complex (Greco et al., 2023; Marasco and Linnemann, 2018; Quan et al., 2013). mTOR (mechanistic target of rapamycin) suppresses autophagy under nutrient-rich conditions, and its inhibition via AMPK (adenosine monophosphate-activated protein kinase) is required for autophagy activation. In T2DM, upregulation of AMPK activity has been shown to inhibit mTOR, reducing autophagic vacuoles and potentially improving β cell function. Exendin-4, an agonist of the glucagon-like peptide GLP-1 receptor (GLP-1R), interacts with most of these intracellular proteins and factors further supporting the idea that autophagy is implicated in T2DM pathogenesis (Masini et al., 2009).

In PD most of these biologic pathways are involved. Under normal conditions, chaperone-mediated autophagy (CMA) degrades α-synuclein by recognizing its pentapeptide sequence. The protein binds to LAMP-2A, a lysosomal membrane protein, and is then translocated into the lysosome for degradation. In PD, defective clearance of α-synuclein due to impaired LAMP-2A function results in dysregulated autophagy. Mutations in LRRK2 impair its function and disrupt the formation of aggressive aggregates (aggresomes) that normally facilitate the autophagic clearance of protein aggregates (Stefanis et al., 2019). PINK1 and Parkin are essential for the clearance of damaged mitochondria via a process known as mitophagy. Under normal conditions, PINK1 accumulates on damaged mitochondria, recruits Parkin, and triggers their elimination by autophagy. In PD, mutations in PINK1 or Parkin disrupt this pathway. Similarly, mutations in p62 result in decreased elimination of misfolded and ubiquitinated proteins (Bang et al., 2016; Yue and Yang, 2013).

In genetic inherited forms of PD such as those linked to *GBA1 gene* mutations, autophagy seems to play a central role in the pathogenesis of disease. *GBA1* encodes for the lysosomal glucocerebrosidase (GCase), an enzyme involved in cellular waste elimination. Clinical alterations resulting from these mutations are hypothesized to be derived from either loss or gain of function of GCase and interaction with its substrates like glycosphingolipids (GSLs), glucosylceramide (GlcCer) and glucosylsphingosine (GlcSph).

The hypothesis of the gain of function in GCase theorizes that the mutant form of protein is recognized as misfolded, thus placing stress on mechanisms of protein degradation and interfering with α-synuclein breakdown and consequent endoplasmic reticulum stress, as observed in autophagy perturbations in cells derived from patients with *GBA1*-associated PD (Fernandes et al., 2016).

#### 2.5.2 Clinical studies

Monogenic PD cases due to *GBA1* loss of function and subsequent substrate accumulation have been investigated in cellular and animal models and in studies of human brains of patients with *GBA1*-associated PD showing to particularly affect pars compacta of SN, putamen, cerebellum, and amygdala and frontal cortex (Gegg et al., 2012; Moors et al., 2019). The accumulation of GlcCer and GlcSph may disrupt autophagy, impair mitochondria, and promote α-synuclein aggregation, although a solid recognized evidence linking decreased GCase activity with substrate accumulation and α-synuclein build-up is still lacking (Rocha et al., 2015).

### 2.6 Oxidative stress

Oxidative stress occurs when the production of reactive oxygen species (ROS) exceeds the ability to neutralize them. While ROS are involved in normal metabolic signaling, excessive ROS can damage lipids, proteins, and DNA, contributing to cellular dysfunction.

#### 2.6.1 Basic science research

Mitochondria are a major source of ROS production during oxidative metabolism, although the overproduction of ROS leads to mitochondrial dysfunction, which is linked to diseases like PD and AD. Additionally, oxidative stress affects endothelial cell function by reducing the availability of nitric oxide (NO), which regulates vascular tone negatively impacting brain health. ROS can also damage oligodendrocytes, leading to myelin loss, which is crucial for proper neuronal communication (Chakrabarti and Bisaglia, 2023; Hassanzadeh and Rahimmi, 2018).

#### 2.6.2 Clinical studies

These mechanisms are involved in MetS as well. Indeed, recent studies using magnetic resonance imaging (MRI) to measure myelin water fraction suggest that obesity, along with conditions like hypertension and metabolic syndrome, is linked to reduced myelin content in the brain (Bouhrara et al., 2021).

### 2.7 Endoplasmic reticulum stress

Endoplasmic Reticulum (ER) stress is a condition that arises from the abnormal buildup of misfolded and unfolded proteins within the ER lumen. It is widely recognized that brief episodes of ER stress trigger the unfolded protein response (UPR), which helps safeguard cells from the harmful effects of misfolded and unfolded protein accumulation. However, prolonged or excessive ER stress cannot effectively be restored by UPR, thus leading to cell death.

#### 2.7.1 Basic science research

The three specific sensor proteins of ER stress are activating transcription factor 6 (ATF6), inositol requiring enzyme 1 α (IRE1α), and eukaryotic pancreatic ER kinase (PKR)–like ER kinase (PERK): each one activates subsets of effector genes comprising ATF4, caspase-12, CHOP, bcl, and NF-κB. When activated in the context of ER stress these factors can lead to β cell apoptosis in T2DM thus contributing to the disease pathogenesis (Fonseca et al., 2011; Hu et al., 2018). Studies have demonstrated the upregulation of ER stress biomarkers in T2DM, and the same set of genes was found to be somehow relevant in PD progression mechanisms. As an example, in a rat model of α-synuclein induced ER stress, in the absence of re-establishment of ER homeostasis, ATF6 activates proapoptotic CHOP protein in DA-ergic neurons. It has also been shown that both HMG-CoA reductase degradation protein-1 (Hrd1) and Parkin-associated endothelin receptor-like receptor (Pael-R) are abnormally expressed in DA-ergic neurons. Hrd1 plays a crucial role in the ER-associated degradation of misfolded or unfolded proteins, while Pael-R acts as a substrate for Parkin. Accumulation of Pael-R causing ER stress could be relevant to the pathological mechanisms that underlie autosomal recessive PD. Inhibition of X-box-binding protein 1 (XBP1), a key regulator of the UPR, was found to induce chronic ER stress and specific DA-ergic neurodegeneration. The restoration of its normal level was linked to decreased striatal denervation and DA-ergic neuronal death in toxic-induced mice model of PD (Valdés et al., 2014; Wang et al., 2015). Therefore, solid evidence growingly indicates that ER stress is a shared pathological characteristic of T2DM and PD, observed in both familial and sporadic models of the latter (Wang D. X. et al., 2020).

### 2.8 Altered iron metabolism

Iron accumulation is reportedly a mechanism that can lead to cellular damage in both neurodegeneration and metabolic dysfunction models, and iron-dependent programmed cell death (ferroptosis) is observed in both conditions (Duţă et al., 2024). Especially in the context of oxidative damage, there are iron dependent mechanisms that contribute to cellular degeneration and α-synuclein aggregation. Fenton reaction (an oxidation process that generates hydroxyl radicals using hydrogen peroxide and ferrous ions) and breakdown of lipid-derived radicals contribute to cellular oxidative stress (Chakrabarti and Bisaglia, 2023).

Ferroptosis has been recently investigated as one of the iron-dependent mechanisms that most intervene in cell death in metabolic dysfunctions and PD. Ferroptosis is a specific iron-dependent programmed cell death morphologically characterized by changes in mitochondria like shrinkage, disappearance of cristae and outer membrane rupture (Dixon et al., 2012; Stockwell et al., 2017). These alterations result from generation of highly reactive free radicals leading to peroxidation of membrane phospolypids rich in polyunsaturated fatty acids, particularly in conditions with increased intracellular iron and decreased antioxidant systems (namely glutathione) (Hirschhorn and Stockwell, 2019).

Iron plays a crucial role in the synthesis of neurotransmitters in the brain such as DA, epinephrine, norepinephrine and serotonine, and in the production of ATP in the mitochondria through the process of electron transport in an organ like the brain that requires a considerable amount of energy. Moreover, it is involved in synthesis of lipid components of myelin and oligodendrocyte development (Zucca et al., 2017). Normally brain concentrations of iron are not influenced by circulating iron levels, due to correct functioning of the blood-brain barrier and blood-cerebrospinal fluid barrier; however when permeability of the barrier with age increases, brain iron accumulation is seen (Farrall and Wardlaw, 2009; Ward et al., 2014). It is hypothesized that in patients with PD, because of an increased proinflammatory state, the blood-brain barrier is even more permeable and as MRI studies show, iron accumulation targets neurons and glia of SN, putamen and globus pallidus (Ward et al., 2014). Other mechanisms that could potentially explain ferric accumulation in PD are increased lactoferrin receptor in neurons and microvessels, increased DMT1 in DAergic neurons, and genetically-determined impaired in iron transport and binding (Faucheux et al., 1995; Mastroberardino et al., 2009; Salazar et al., 2008).

Neuromelanin, an oxidation-derived pigment from DA, has been reported to fuel inflammation in certain conditions such as in PD through microglia activation. Besides DA, neuromelanin also contains metals like iron. The accumulation of iron can promote neuromelanin formation, but on the other hand, neuromelanin reduces the potential damage driven by iron to other cells by trapping it (Wise et al., 2022). In general, conditions that lead to excessive iron storage, via oxidative pathways, can cause organ damage. For example, this promotes fibrogenesis in the liver worsening the course of underlying hepatic disorders, and a similar worsening effect is observed on the course of other chronic disorders apparently not directly linked to iron homeostasis such as metabolic and cardiovascular disorders (Pietrangelo, 2016).

#### 2.8.1 Basic science research

Nigral iron deposition is demonstrated in in-vivo rat models of PD (Berg et al., 1999) and some evidences also show an alteration in iron metabolism in Zona Incerta, a brain region recently implicated in PD pathology in models of MPTP- and 6-OHDA-induced PD in mice (Xiu et al., 2025).

The effect of altered iron homeostasis on metabolic impairments has also been studied in mouse models. There is evidence that iron accumulation or deficiency can alter adipose tissue microenvironment and secretion of IR-related adipokines (leptin and adiponectin), and lead to reduced glucose transport after insulin stimulation (Huang et al., 2011). Viceversa, in mice fed a high-fed diet inducing weight gain, hepatic steatosis and IR were accompanied by alterations in expression of hepcidin (a protein that regulates iron homeostasis in response to erythropoietic activity) which preceded dysregulation of iron concentrations particularly in liver and visceral adipose tissue, with no effect on serum iron levels (Varghese et al., 2020). Therefore, metabolic pathways of iron, glucose and fat seem to interplay with each other both in a physiological and pathologic status.

#### 2.8.2 Clinical studies

Increased accumulation of iron in substantia nigra of PD patients was observed with MRI techniques in post mortem studies with significant accumulation in putamen, red nucleus, nucleus caudatus and globus pallidus (Wang et al., 2016) and similar imaging findings on nigrosome imaging were found in cases of PD and parkinsonian syndromes (Bae et al., 2021). Curiously, some data seem to suggest that although iron deposition measured via magnetic susceptibility occurs early in PD, the amount of iron does not differ in groups of different disease stages (Li et al., 2022). Addingly, another study not only showed increased iron concentration in SN pars compacta of early-stage PD patients compared to controls, but in those with late-stage PD apart from affecting SN pars compacta, other areas such as red nucleus and globus pallidus became involved suggesting a regionally progressive accumulation that is consistent with the stage of the disease (Guan et al., 2017). A recent review taking into account studies on non-invasive neuroimaging highlighted the utility of quantitative magnetic susceptibility mapping which allows *in vivo* assessment of iron dysregulation and supports the thesis that region-specific brain iron accumulation could represent a valuable biomarker in diagnosis, prognosis and disease monitoring in PD and parkinsonism (Guan et al., 2024).

Potential association between iron metabolism and T2DM pathobiology has been studied *in vivo* on a Chinese population of T2DM of middle-aged and elderly patients with evidence of a linear correlation between ferritin levels and risk of developing T2DM (Sun et al., 2013). Reducing iron storage levels *in vivo* via phlebotomies has resulted in improved insulin secretion and peripheral tissue insulin sensitivity, consequently leading to better control of blood glucose and T2DM condition improvement (Fernández-Real et al., 2002).

In obesity, iron levels are known to be low and in iron deficiency rate increases with BMI. This is partly due to altered micronutrients intake (Nead et al., 2004; Pilar Vaquero et al., 2021); however higher hepcidin levels are registered in this population, making low serum iron not only dilutional or nutritional, but linking it to chronic systemic low grade inflammation (Tussing-Humphreys et al., 2010). Studies showed that transferrin saturation is negatively influenced by BMI and waist circumference (Pilar Vaquero et al., 2021; Stoffel et al., 2020).

### 2.9 Gut microbiome alteration

There is still much controversy as to what constitutes a “healthy” microbiome and it is difficult to classify bacteria as either “good” or “bad”. In general, bacteria associated with a healthy microbiome include those that produce short-chain fatty acids (SCFAs) such as acetic acid, propionic acid, and butyric acid, with an overall positive impact on the integrity of the intestinal barrier (Lee et al., 2017; Naghipour et al., 2024; Van Hul and Cani, 2023). Genera considered harmful in the microbiome include potential pathogens and bacteria that produce toxins, such as lipopolysaccharide (LPS). Gut-derived LPS, due to the disrupted function of the intestinal barrier and increased permeability in PD, is likely to enter the bloodstream and cause systemic inflammation. It can also cross the blood–brain barrier and bind to toll-like receptor 4 on microglial cells, thus promoting TNF-α, interleukin (IL)-1β, and IL-6 expression and DA-ergic neuron damage. It is important to remember that a healthy microbiome is associated with high microbiota diversity and a balanced bacterial composition (Bicknell et al., 2023). The gut–brain axis refers to the intricate, bidirectional communication network between the CNS and the gut, involving a complex interplay of neural, hormonal, and immune signaling mechanisms. Metabolites produced by the microbiome can directly affect the mitochondria in neurons and brain metabolism.

#### 2.9.1 Clinical studies

The hypothesis that gut diseases influence the CNS is supported by the finding that colon biopsies from patients with PD display higher CD3 + T cell infiltration, upregulated expression of the proinflammatory cytokines interleukin-1β (IL-1β) and interferon-gamma, and elevated calreticulin levels in mucosal tissue and feces. Gut barrier permeability dysfunction in PD is also supported by the reduced expression levels of the tight junction proteins Zonula Occludens-1 (ZO-1) and occludin in colonic mucosal samples from patients (Clairembault et al., 2015). Many PD patients may exhibit digestive symptoms years before the onset of neurological symptoms, and the gut microbiome composition in PD patients differs significantly from that of healthy controls (Fan et al., 2022; Hirayama and Ohno, 2021). Animal models of PD have shown that an altered microbiome can lead to a build-up of α-synuclein in the gut, which is then transported to the brain. A similar mechanism has been suggested in humans, and studies on a population after vagotomy found vagus nerve truncation to be protective against the onset of PD (Svensson et al., 2015). Dysregulation of TLR signaling, stimulated by gut dysbiosis and subsequent local inflammation, may trigger α-synuclein aggregation. A relevant aspect is how environmental exposures may interact with the gut microbiome to influence PD. For example, MetS is associated with alterations in the gut microbiome (Dabke et al., 2019). The liver may also contribute to the effects of the gut microbiome on the brain through the gut–liver–brain axis (Ding et al., 2020). Furthermore, obesity-associated gut dysbiosis has been linked to the release of various bacterial toxins into the bloodstream, which can exert an influence on the CNS (Breton et al., 2022). On the other hand, PD has been observed able to facilitate intestinal glucose metabolism disorders via autonomic disfunction and microbial alteration such as the increase in *Pseudoflavonifractor*, bacteria associated with impaired energy metabolism and insulin sensitivity found over-represented in PD patients gut (Kim et al., 2022; Wang Y. et al., 2020).

Multiple clinical studies have demonstrated an altered microbiome (dysbiosis) in PD. The PD microbiome shows reduced α-diversity compared to healthy controls. The genera most commonly found to be decreased or underrepresented in the microbiota of PD are the beneficial bacteria (SCFA-producing and anti-inflammatory), including *Faecalibacterium*, *Roseburia*, *Ruminococcus*, *Prevotella*, *Dorea*, *Bacteroides*, *Clostridium* cluster IV (leptum), and genera in the order *Lachnospirales*. Bacteria that are overrepresented in PD include *Akkermansia*, *Bifidobacterium*, and *Lactobacillus*, and opportunistic/potential pathogens *Enterococcus* (endotoxin-producing) *Christensenella*, *Oscillospira*, *Corynebacterium*, *Alistipes*, some *Bacteroides*, *Megasphaera*, *Desulfovibrio*, *Streptococcus*, *Staphylococcus*, and the family Enterobacteriaceae (*E. coli/Shigella*, *Salmonella*, *Klebsiella*), *Helicobacter pylori*. A number of these bacteria produce LPS and other bacterial toxins, which increase pro-inflammatory cytokines when passing into the tissues. *Akkermansia* is thought to promote barrier degradation because it utilizes the mucus layer as an energy source (Heintz-Buschart et al., 2018). In a pro-inflammatory environment, the formation of aggregated α-synuclein and the disruption of the brain-blood barrier is promoted. Increases in Enterobacteriaceae have been associated with motor symptom progression in PD. It is somewhat paradoxical that *Akkermansia*, *Bifidobacterium*, and *Lactobacillus* are often found to be increased in PD and would, in other circumstances, be recognized as beneficial bacteria and are marketed as probiotics (Bicknell et al., 2023; Fan et al., 2022; Hirayama and Ohno, 2021; Wang et al., 2025). It was reported that probiotic supplementation with *Lactobacillus acidophilus*, *Bifidobacterium bifidum*, *L. reuteri*, and *L. fermentum* improved motor scores in PD patients. Additionally, in mouse models of PD, transplanting *E. faecalis* and *E. faecium* into PD mouse models significantly increased dopamine levels in the brain and improved motor deficits (Wang et al., 2025).

## 3 Prevention and treatment interventions

### 3.1 Lifestyle and dietary interventions

Considering the lack of current treatments for PD beyond symptom management, and the growing evidence of MetS and NBD being intertwined, lifestyle and dietary modifications have been taken into account as a potential part of the treatment.

#### 3.1.1 Clinical studies

A recently published meta-analysis of 12 studies on the adherence to the Mediterranean diet and the incidence of PD indicated that the highest adherence to the Mediterranean diet showed a significant negative correlation with the incidence of PD, with an overall OR of 0.75 (95% CI: 0.66, 0.84) and an even more accentuated effect on the prodromal phase of the disease with an OR of 0.67 (95%CI: 0.59, 0.76) (Zhao et al., 2025). A pilot randomized clinical trial (RCT) testing PD patients to maintain a low-fat or ketogenic diet for 8 weeks observed that both groups significantly improved in motor and nonmotor symptoms, but the ketogenic group showed greater improvements in nonmotor symptoms (Phillips et al., 2018). A review on the effect of different types of diet on PD pointed out that a vegan diet through the increased intake of vegetables, nuts, and tea correlated with a reduced PD risk (28, 31, and 25%, respectively), likely due to protection by antioxidant-rich functional foods containing vitamins C and E, polyphenols, and carotenoids and antinflammatory compounds like flavonoids. A possible mechanism explaining this is that a plant-based diet low in certain proteins may elevate the levels of Parkin and the protective PINK1 kinase. As for the carnivore diet, based on consumption of animal products, with the elimination or minimal consumption of plant-based foods, it is known that total consumption of red meat is positively associated with the onset of MetS and elevation in LDL cholesterol. Although some studies correlate higher serum LDL levels with a lower risk of PD, these findings are still indeterminate (Ansari et al., 2024).

In a cohort study on 4000 identified cases from the UK Biobank, higher levels of physical activity were found to be significantly associated with a reduced risk of developing PD (Q3: HR 0.86; Q4: HR 0.81), and the effect was even more pronounced in individuals who adhered to a plant-based diet (Zheng et al., 2024).

Neuroplastic changes induced by physical exercise have been thought to mainly involve the striatum and prefrontal cortex, as well as modulation in the release of DA, cytokines, and neurotrophic factors (such as the brain-derived neurotrophic factor), increased corticomotor excitability, improved connectivity in sensorimotor networks, and changes in DA receptor activity (Kaagman et al., 2024).

Hence, both exercise, especially moderate anaerobic physical exercise, and a healthy diet rich in plant-derived nutrients, can positively affect PD incidence and progression, by stimulating neurotrophic factors, altering the diversity of the gut microbiome, improving barrier permeability, and increasing the levels of SCFAs- producing bacteria (Raber and Sharpton, 2023).

### 3.2 Antidiabetic treatments

An Italian, single-center retro- and prospective study analyzed the age of onset of PD in a cohort of patients in relation to the onset of T2DM (Pezzoli et al., 2023). It was observed that diabetic patients, regardless of the type of antidiabetic therapy, developed PD more than 6 years later than non-diabetic subjects or those who developed diabetes after the onset of PD.

This delay in PD onset represents around 30% of a typical Parkinson’s patient’s life expectancy, which may contribute to lower prevalence of diabetes in the PD population, as some diabetic patients may not live long enough to develop PD. The study suggests that antidiabetic drugs might be linked to a delayed onset of PD (Pezzoli et al., 2023).

#### 3.2.1 Metformin

##### 3.2.1.1 Basic science research

Despite metformin being the most widely prescribed drug for the treatment of T2DM and being considered an “essential medicine” by the World Health Organization, its exact mechanism of action is still debated. It is thought that its primary action is on the liver and it was demonstrated that it inhibits hepatic gluconeogenesis both *in vitro* and *in vivo* in a redox-dependent manner without concomitant increases in plasma insulin concentrations. Peripheral and intestinal glucose metabolism have also been shown to be positively affected by metformin. Gluconeogenesis accounts for the most consistent part of the total hepatic glucose production (together with glycogenolysis, glycogen synthesis, and glycolysis), therefore it represents an important pharmacologic target. Postulated mechanisms of metformin action, including AMPK activation and complex I inhibition, have been demonstrated in the setting of a consistent pleiotropic activity of this drug, suggesting that several molecular targets could be involved (LaMoia and Shulman, 2021). Recent studies show that metformin can alleviate age-associated inflammation by increasing autophagy and improving mitochondrial function, both of which are relevant biological features involved in PD-related cellular damage pattern (Bharath et al., 2020). Several studies have now explored the capacity of metformin to suppress ER stress and prevent ER stress-induced apoptosis through AMPK-PI3K-c-Jun NH2 pathway (Jung et al., 2012), a mechanism that can protect pancreatic β cell against lipotoxicity. There is also evidence that metformin protects against MPTP-induced neurotoxicity, likely by inhibiting α-synuclein phosphorylation, inducing neurotrophic factors, and promoting antioxidant activity (Patil et al., 2014; Paudel et al., 2020). Similar neuroprotective effects have been observed in mouse models of rotenone-induced PD as well, through the inhibition of α-synuclein phosphorylation and aggregation, protection of mitochondrial function, inhibition of neuroinflammation, modulation of autophagy, attenuation of oxidative stress and restoration of ER balance (Wang D. X. et al., 2020).

##### 3.2.1.2 Clinical studies

Based on the mechanisms described so far, the repurposed use of metformin in NBD may offer promising opportunities. Some epidemiological studies investigated in cohorts of T2DM patients the correlation between metformin therapy and the risk of PD. A longitudinal study analyzed a 5-year follow-up of more than 5500 U.S. veterans with T2DM, reporting that metformin therapy lasting more than 4 years significantly decreased the risk of developing both PD and AD (Shi et al., 2019). On the other hand, a systematic review and meta-analysis by Qin et al. (2021) showed a lack of correlation between metformin therapy and PD development (HR 1.23, 95% CI 0.98–1.78). Furthermore, after the exclusion of a single study, results indicated a significantly increased PD risk with the use of metformin (HR, 1.50; 95% CI, 1.11 –2.02). These conflicting results may derive from a high level of heterogeneity across clinical studies, including variations in population, treatment protocols, follow-up durations, and adjustment factors.

Recent research suggests that metformin may also alter the gut microbiota, through the protection of the intestinal barrier, promotion of SCFAs producing bacteria growth, regulation of bile acids plasmatic levels and their excretion, and direct regulation of growth of bacteria with a positive effect on glucose homeostasis (eg., *Akkermansia muciniphila*) (Zhang and Hu, 2020).

##### 3.2.1.3 Comment

In conclusion, high-quality studies are needed to determine the potential role of metformin in PD, focusing on its precise mechanism of action and the elucidation of its double-edged effects.

#### 3.2.2 Incretin mimetics

The studies using insulin showed promising results. However, insulin is not an ideal drug to be developed as a major treatment for PD due to insulin desensitization. Research of novel drug treatments for diabetes has thus moved away from insulin analogs to new peptide hormones that have similar effects, in particular the incretin hormones, such as Glucagon-like peptide-1 (GLP-1), glucose-dependent insulinotropic polypeptide (GIP) and Dipeptidyl peptidase-4 (DPP-4), a key enzyme responsible for cleaving and inactivating both of GLP-1 and GIP peptides at the penultimate alanine residue (Drucker, 2006). GLP-1 and GIP are secreted by specific intestinal enteroendocrine cells in response to nutrient ingestion and absorption, preferably of carbohydrates and fat. These hormones promote pancreatic β cell proliferation and differentiation, as well as the induction of glucose-dependent pancreatic insulin secretion. Additionally, GLP-1 acts as a glucagon inhibitor in a postprandial state, while GIP stimulates glucagon release during fasting. Incretin hormone receptors are expressed not only peripherally but also in the CNS, thus potentially restoring brain insulin sensitivity by activating insulin-mediated pathways that promote neuron survival, while inhibiting pro-apoptotic pathways (Drucker, 2006).

GLP-1 is part of the peptide growth-factor family and activates a glucagon-type seven membrane-spanning G-protein coupled receptor. GLP-1 receptors are expressed in neurons of rodents, primates, and humans (Cork et al., 2015; Farr et al., 2016; Heppner et al., 2015; Merchenthaler et al., 1999). GIP receptor expression has been found in large neurons such as the pyramidal neurons in the cortex and hippocampus, granule neurons in the dentate gyrus, Purkinje cells in the cerebellum, and basal brain areas (Nyberg et al., 2005).

In the last few years, a group of new medications for T2DM was developed, including GLP-1 and GIP analogs. These drugs do not enhance insulin desensitization; instead, they can resensitize insulin signaling. In addition, GLP-1 analogs do not affect blood glucose levels in normoglycemic people and therefore can be safely given to non-diabetic PD patients. The side effects are very mild and include nausea and loss of appetite. Targeting the catalytic action of DPP-4 with inhibitors is also effective in the treatment of T2DM. All together, these drugs can be grouped under the name of GLP-modulating agents (GLP-MA).

##### 3.2.2.1 Basic science research

In animal models of PD, GIP analogs showed promising results with improvement in motor symptoms and reduction of the inflammation response (Li et al., 2016). Greig and colleagues were the first to test GLP-1 analogs in preclinical studies to discover that they have neuroprotective properties (Perry and Greig, 2004; Perry et al., 2002). Further work showed that GLP-1 receptor agonists have neuroprotective effects in animal models of AD and PD and re-sensitize insulin signaling (Airaksinen and Saarma, 2002; Cao et al., 2016; Feng et al., 2018; Hölscher, 2018; Jalewa et al., 2017; Ji et al., 2016; Vaccari et al., 2021)An extensive systematic review and meta-analysis including preclinical studies on the effects of GLP-1 agonists, DPP-4 inhibitors, and dual GLP-1/GIP agonists on NBD, found that liraglutide (Hansen et al., 2016) and saxagliptin (Turnes et al., 2018), tested on moderate to severe DA-ergic neuron loss, were not effective in reversing the effects of 6-OHDA on DA-ergic neurons and associated behaviors. Exendin-4 (a peptide that shares 53% amino acid sequence with native GLP-1), however, was found to be effective in rat models for preventing 6-OHDA-induced DA-ergic neuron loss and increasing the number of neural stem cells in the subventricular zone (Bertilsson et al., 2008). In other models of MPTP-induced PD, the effect of DPP-4 inhibitors was found to be superior compared to exendin-4. In general, GLP-MA were found to be not as effective in reversing the impairments induced by MPTP models as 6-OHDA models (Erbil et al., 2019). Another recently investigated agent is tirzepatide, a peptide with dual GIP and GLP-1 receptor coagonist activity with a bias toward GIP receptor, that was reportedly protective against rotenone-induced PD in rats. In a recent study it restored behavioral deficits and modulated oxidative stress markers by upregulating glutathione and superoxide dismutase concentration (both with a protective antioxidant effect) and downregulating malondialdehyde (a lipid peroxidation marker) and oxidized glutathione. Moreover, tirzepatide restored the striatal dopamine level previously diminished by rotenone and reduced the levels of TNF-α and IL-6. Lastly, it reduced α-synuclein hyperphosphorylation in the pars compacta of SN (Delvadia et al., 2025). Comprehensively, at the preclinical level, the molecular mechanisms through which GLP-MA exert their positive effects on neuroprotection can be summarized in the reduction of inflammation, increase of anti-apoptotic proteins neural stem cells in the subventricular zone, strengthening of the blood-brain barrier, changes in the phosphorylation levels of molecules as GSK-3b, Akt, Erk and mTOR, and modulation of the glutamate system, NMDA receptors and calcium signaling (Erbil et al., 2019).

##### 3.2.2.2 Clinical studies

At the clinical level, RCTs have been carried out to evaluate the effects of some GLP-MA in PD patients (Table 2) (Hölscher, 2024). A phase II study of liraglutide in addition to DRT showed a significant improvement in motor and non-motor symptom scores (Hogg et al., 2023). Similarly, in a phase II trial on lixisenatide, PD patients in the treatment group after 12 months registered a significant (*p* = 0.0068) primary outcome endpoint change in motor tests, and the positive effect persisted at 2 months after discontinuation of treatment (*p* < 0.05) (Meissner et al., 2024). In a recent work by Athauda et al. (2017), exenatide was tested in an RCT involving PD patients. Positive effects on practically defined off-medication motor scores were observed compared to placebo, which were sustained beyond the period of exposure. However, in a recently published phase 3, multicentric, double-blind, parallel-group, randomized, placebo-controlled trial (Vijiaratnam et al., 2025), the off-medication scores at 96 weeks had worsened by a mean of 5.7 points in the exenatide group and by 4.5 points in the placebo group, thus failing to support the role of exenatide as a disease-modifying treatment for PD patients. In an RCT using NLY01, a brain-penetrant, pegylated, longer-lasting version of exenatide on PD patients, the primary outcome was negative for improvement in PD motor or non-motor features versus placebo, although a subgroup analysis raised the possibility of motor benefit in younger participants (McGarry et al., 2024).

**TABLE 2:** Summarization of the clinical studies assessing efficacy of drugs of the class of GLP-MA on PD evolution.

Year	Author	Phase	Design	Drug	*n*	Target population	Outcome
2022	Hogg et al., 2023	Phase II	Single-center, randomized, Double-Blinded, Placebo-Controlled Trial	Liraglutide	*N* = 63	PD patients with at least 2 years disease duration or with positive Ioflupane I123 DaT Scan, with responsiveness to DRT	Treatment with liraglutide is safe and improves critical features of PD, including non-motor symptoms, overall mobility, activities of daily living, and quality of life
2024	Meissner et al., 2024	Phase II	Multicenter, double-blind, randomized, placebo-controlled trial	Lixisenatide	*N* = 156	Early diagnosed (<3 years) PD patients on DRT for at least 1 month	Lixisenatide therapy resulted in less progression of motor disability than placebo at 12 months but was associated with gastrointestinal side effects
2017	Athauda et al., 2017	Phase II	Single center, randomized, double-blind, placebo-controlled trial	Exenatide	*N* = 62	PD patients on DRT with wearing-off effect	Exenatide had positive effects on practically defined off-medication motor scores in PD, which were sustained beyond the period of exposure
2025	Vijiaratnam et al., 2025	Phase III	Multicentre, double-blind, parallel-group, randomized, placebo-controlled trial	Exenatide	*N* = 194	PD patients on DRT	No evidence to support exenatide as a disease-modifying treatment for people with PD
2024	McGarry et al., 2024	Phase II	Randomized, double-blind, placebo-controlled trial	NLY01 (brain-penetrant pegylated analog of exenatide)	*N* = 225	Patients with early untreated PD	NLY01 was not associated with improvement in PD motor or non-motor features compared to placebo. Subgroup analysis suggests possibility of motor benefit in younger patients.

##### 3.2.2.3 Comment

Taken together, findings from preclinical and clinical studies show that GLP-MA may hold the potential for alleviating motor and non-motor symptoms in PD. However, additional research, including larger and longer-term clinical trials, is needed to fully assess their efficacy and safety in the context of PD.

## 4 Conclusion and perspective

In conclusion, this review considers the growing emerging evidence linking PD to clinical entities like obesity, T2DM and MetS grouped under a metabolic dysfunction continuum. Therefore, the possibility of PD being a manifestation of said altered metabolic spectrum progressively surfaced.

At a preclinical level, similar molecular mechanisms at the basis of cellular injury in these conditions explain the shared pathways of biological damage suggesting that a common pathogenesis is possible and that it is mainly driven by IR, pro-inflammatory environment and oxidative stress. Consequently, pharmacologic approaches to PD models with drugs originally created for T2DM have been tested and showed protective effect toward the development of cellular damage associated with PD. Results from clinical research were adherent to the hypothesis that that PD is more represented in patients with a higher metabolic burden, and clinical studies confirmed the benefit of metformin and some GLP-MA on PD-related outcomes.

In addition, gut microbiota is increasingly investigated as a marker of brain health considering that the spectrum of bacteria populating bowel in these pathologic conditions shows under- or overrepresentation in genera and species that significantly differ from non-pathological models. Although these findings seem solid, gut-brain axis is continuously under study for better comprehension of its role and potential therapeutical interventions.

However, a solid knowledge on how neurologic and metabolic molecular pathways interplay in the complexity of the human model is still lacking. Reasons at the basis of this issue are not trivial. The absence of prodromal markers of PD presumably impacts negatively on the possibility of studying early metabolic features associated with the disease, nor does it facilitate the detection of at-risk patients that could benefit a metabolic screening and intervention. On the other hand, once PD progresses to a late stage, the disease-related impairments could represent a detriment to the metabolic homeostasis and its evaluations. Data on genetic patterns associated with PD and impaired metabolism also lack. In a future prospective, it would be ideal to define a model that combines prodromal manifestations of PD and metabolic dysfunctions that is also aimed to untangle the genetic signature behind the multiple faces of this neurologic and systemic ensemble.
